# In-depth first-principle study on novel MoS_2_ polymorphs†

**DOI:** 10.1039/d0ra10443d

**Published:** 2021-01-19

**Authors:** Håkon Eidsvåg, Murugesan Rasukkannu, Dhayalan Velauthapillai, Ponniah Vajeeston

**Affiliations:** Department of Computing, Mathematics and Physics, Western Norway University of Applied Sciences Inndalsveien 28, Box 5063 Bergen Norway heid@hvl.no; Center for Materials Science and Nanotechnology, Department of Chemistry, University of Oslo Box 1033 Blindern N-0315 Oslo Norway

## Abstract

Molybdenum disulphide (MoS_2_) is a rising star among transition-metal dichalcogenides in photovoltaics, diodes, electronic circuits, transistors and as a photocatalyst for hydrogen evolution. A wide range of MoS_2_ polymorphs with varying electrical, optical and catalytic properties is of interest. However, in-depth studies on the structural stability of the various MoS_2_ polymorphs are still lacking. For the very first time, 14 different MoS_2_ polymorphs are proposed in this study and in-depth analysis of these polymorphs are carried out by employing first-principle calculations based on density functional theory (DFT). In order to investigate the feasibility of these polymorphs for practical applications, we employ wide range of analytical methods including band structure, phonon and elastic constant calculations. Three of the polymorphs were shown to be unstable based on the energy volume calculations. Among the remaining eleven polymorphs (1T_1_, 1T_2_, 1H, 2T, 2H, 2R_1_, 2R_2_, 3H_a_, 3H_b_, 3R and 4T), we confirm that the 1T_1_, 1T_2_, 2R_2_ and 3R polymorphs are not dynamically stable based on phonon calculations. Recent research suggests that stabilising dopants (*e.g.* Li) are needed if 1T polymorphs to be synthesised. Our study further shows that the remaining seven polymorphs are both dynamically and mechanically stable, which make them interesting candidates for optoelectronics applications. Due to high electron mobility and a bandgap of 1.95 eV, one of the MoS_2_ polymorphs (3H_b_-MoS_2_) is proposed to be the most promising candidate for these applications.

## Abstract 2

14 new MoS_2_ polymorphs were studied using first-principle calculations based on density functional theory. We found a new promising MoS_2_ candidate for photocatalytic and photovoltaic applications.

## Graphical abstract

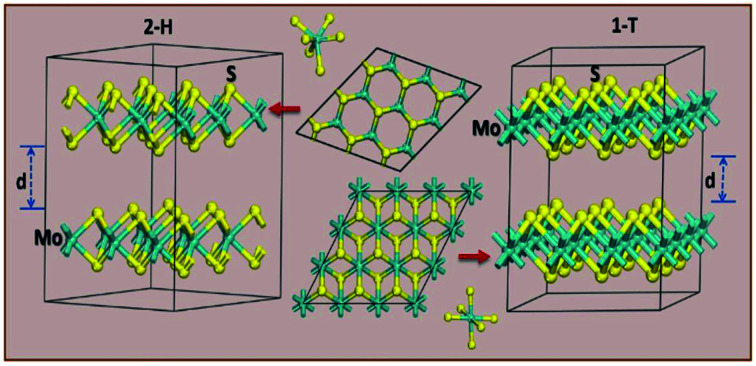

## Introduction

Recent research has established transition-metal dichalcogenides (TMDs) as a promising material within several fields.^1^ This is due to their unique optical, electronic and structural properties, which are dependent on the layered structure of the TMDs.^2–4^ Molybdenum disulphide (MoS_2_) is perhaps the most well-known TMD with an indirect electronic bandgap of 1.2 eV (experimental value for bulk MoS_2_),^5^ which is surprising as it has a graphene-like polymorph. This is mainly because the electronic properties for TMDs are based on filling the d orbitals, in contrast to graphene and silicon where it is the hybridization of s and p orbitals that lays the foundation for the electronic properties.^6^ In addition to the low bandgap, MoS_2_ is a low-cost material; it has a high surface-to-volume ratio and an abundance of active sites making it attractive in several fields.^7^ Currently, MoS_2_ is known for its properties as a lubricant^8^ and lately in photovoltaic (PV) cells,^9^ as a photocatalyst for hydrogen evolution,^10^ as gas or biosensors^11,12^ and as a transistor that can operate at room temperature.^4^ Especially within photocatalytic water splitting MoS_2_ is seen as the potential successor to TiO_2_ photocatalysts due to the tuneable bandgaps, its high charge carrier mobility, high transparency and excellent chemical stability.^7^

The MoS_2_ polymorphs consists of a layer of Mo (transition metal) sandwiched between two layers of S (chalcogens) and strong covalent bonds are present within the sandwich, while the interlayer bonds between two layers are van der Waals bonds.^13^ Depending on the coordinate configuration MoS_2_ can exist in different phases including 2H, 3R, 1T, 1T′, 1T′′, *etc.*^14,15^ Amongst these, two phases stand out in terms of favourable structural properties: 2H MoS_2_ is a thermodynamically stable phase with A–B–A sandwich layers that occurs at ambient pressure conditions, this is also the most commonly used phase. 1T is a metastable phase, A–B–C layers, and has not been strictly determined due to a lack of a strict structural refinement. Another important distinction between the two phases is that 1T is metallic while 2H is a semiconductor/insulator.^11^

An alternative to adding dopants to transform materials from insulators to metals is utilising high pressure during the synthesis. It is well known that bilayer sheets of MoS_2_ go through a semiconductor–metal transition upon vertical compressive pressure. Early research suggests that bulk MoS_2_ could metallize under pressure as they found that the bandgap shrinks due to a negative pressure coefficient of resistivity, d*E*_G_/d*P* < 0.^16^ Unfortunately, the structural transition is unknown, and it requires further research.

Most of the work done on MoS_2_ by the research community so far is experimental with focus on synthesis, characterization and application of the material as a photocatalyst.^7,11,12,17–20^ However, over the last years, we have seen a rise in computational work,^21^ including a pioneering work by Byskov *et al.*^22^ As the MoS_2_ structure can easily be modified by changing the stacking sequence and/or the layer distance, a variety of MoS_2_ polymorphs could be synthesised. However, a fundamental understanding of how these modifications will affect the structural stability of the material is still lacking. This knowledge is of utmost importance as different configurations have different properties, making them viable for a diverse range of applications. So far, the challenges have been to synthesise MoS_2_ polymorphs and to identify the stacking sequences. In this study, we propose as many as 14 different MoS_2_ polymorphs and carry out in-depth theoretical analysis on their properties based on DFT calculations. We verify analytically how the different layers and coordinate configuration of MoS_2_ affect the stability and electronic properties of the bulk material. For the very first-time, direct comparison between calculated Raman and IR spectra for pure 1T-MoS_2_ and 2H-MoS_2_. The main objective of this study is to explore the possible metastable phases of MoS_2_ and their relative stability.

## Method of calculations

All the calculations were performed within the periodic density functional theory framework, as it is implemented in the VASP code.^23–27^ The interaction between the core (Mo: [Kr] 4d^5^ 5s^1^, and S: [Ne] 3s^2^ 3p^4^) and the valence electrons were described using the projector-augmented wave (PAW) method.^26,28^ In order to speed up our structural optimisation process, the initial structures were optimised with the Perdew–Burke–Ernzerhof (PBE) exchange–correlation functional.^27^ The obtained PBE level optimised structures were further optimised with the DFT/vdW-DF2 method and based on this, the energy volume curves were generated.^29–31^ Our previous calculations suggested^32^ that structural parameters in oxides could be reliably predicted only by using large energy-cut off to guarantee basis-set completeness. Hence, we have used a cut-off of 600 eV. The atoms were deemed to be relaxed when all atomic forces were less than 0.02 eV Å^−1^ and the geometries were assumed to get optimized when the total energy converged to less than 1 meV between two consecutive geometric optimization steps. The electronic properties were computed by using the screened hybrid functional as proposed by Heyd, Scuseria and Ernzerhof (HSE06) for the polymorphs optimized at the PBE level.^33^ If not specified differently, we used a Monkhorst–Pack 9 × 9 × 9 *k*-mesh for the structural optimization and the electronic polymorph studies. Band polymorphs were computed by solving the periodic Kohn–Sham equation on ten *k*-points along each direction of high symmetry of the irreducible part of the first Brillouin zone.

The supercell method is used for phonon calculations.^34^ The VASP code is used to calculate the real space force constants of supercells, and the PHONOPY^35^ code is used to calculate the phonon frequencies from the force constants on a supercell consisting of at least 32 atoms in all systems. In order to get the force-constant matrices for each binary system, every atom is displaced by a finite displacement of 0.01 Å in *x*-, *y*- and *z*-direction. Strict energy convergence criteria of (10^−8^ eV) and 4 × 4 × 4 *k*-points were used for the force constant calculations. After getting the force-constant matrices, the dynamical matrix is built for different **q** vectors in the Brillouin zone. The eigenvalues of phonon frequencies and eigenvectors of phonon modes are found by solving the dynamical matrix. The thermodynamic properties require summations over the phonon eigenvectors which is implemented in the PHONOPY code. We have checked the dynamical stability of all systems, and no imaginary modes are observed in the polymorphs. The thermal properties, including heat capacity, free energy and entropy, were obtained from the calculated PhDOS. The phonon band polymorphs figures for all the studied systems have also been added to the ESI under SI3 and SI4.† Our study is then completed by evaluating the mechanical stability by computing the single-crystal elastic constants. A set of strains (−0.015 −0.010 −0.005 0.000 0.005 0.010 0.015) is applied to the crystal cell, and the stress tensor is calculated. The elastic constants are then evaluated by linear fitting of the stress–strain curve using VASPKIT.^36^

## Results and discussion

### Structural stability and optimization

**Fig. 1:**
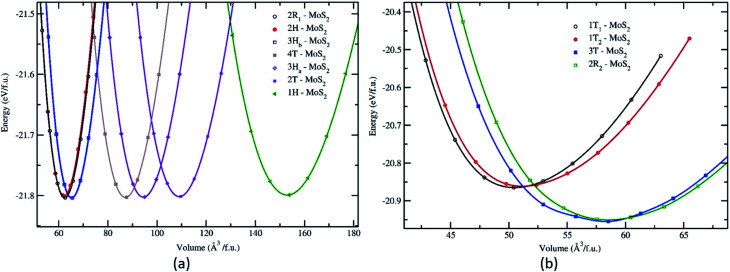
Calculated total energy as a function of the volume of the unit cell for the different phases and polymorphs of MoS_2_. The total energy *vs.* volume curve for the group A (a) and group B (b). All the energy volumes are normalized to one formula unit (f.u.).

Structurally, MoS_2_ can be regarded as strongly bonded two-dimensional S–Mo–S layers or sandwiches which are loosely coupled to one another by relatively weak van der Waals-type forces. Within a single S–Mo–S sandwich, the Mo and S atoms create two-dimensional hexagonal arrays. Depending on the relative alignment of the two S-atom sheets within a single S–Mo–S sandwich, two distinct two-dimensional crystal polymorphs are obtained. In one, the metal atoms are octahedrally coordinated by six neighbouring S atoms, whereas in the other, the coordination of the metal atoms is trigonal prismatic. Variations in the stacking sequence and registry of successive S–Mo–S sandwiches along the hexagonal *c* axis lead to a large number of crystal polymorphs or polytypes in three dimensions. These are referred to as the 1T, 2H, 3R, 4H_a_, 4H_b_ and 6R phases. In this abbreviated notation, the integer indicates the number of S–Mo–S sandwiches per unit cell along the hexagonal *c* axis and T, H, and R denote trigonal, hexagonal, and rhombohedral symmetries, respectively. Variations in the stacking sequence like A, A′, B, B′, C, C′′, *etc.* (for more details see Fig. 3) and variation in the layer–layer distances means we can tune these compounds into several modifications. In order to understand the relative stability of these modifications, we have considered the following 14 polymorphs and they have been used as starting inputs in the structural optimization calculations (number of formula units; and Materials Project ID are given in parenthesis; low energy polymorph identified this work is highlighted as bold letters): *R*3*m* (1, 1434, **2R**_**1**_), *P*3̄*m*1 (4, 1027525, **4T**), *P*6_3_/*mmc* (2, 2815, **2H**); *P*6̄*m*2 (3, 1025874, **3H**_**a**_), *P*3̄*m*1 (3, 1023939, **2T**), *P*6_3_/*mmc* (6, 1018809, **3H**_**b**_), *P*6̄*m*2 (1, 1023924, **1H**), *Pmmn* (18, 990083), *F*4̄3*m* (4, 11780), *R*3̄*m* (1, 558544, **3T**), *P*3̄*m*1 (1, 1238797, **2R**_**2**_), *I*4̄2*d* (2, 1042086), *P*3̄ (1, **1T**_**2**_) and *P*3̄*m*1 (1, **1T**_**1**_). However, the following polymorphs *Pmmn* (18, 990083), *F*4̄3*m* (4, 11780) and *I*4̄2*d* (2, 1042086) are omitted from the rest of the analysis. This is because their energy-volume data are far away from the others as presented in Fig. 1, they are also unstable compared to the other polymorphs.

**Fig. 2:**
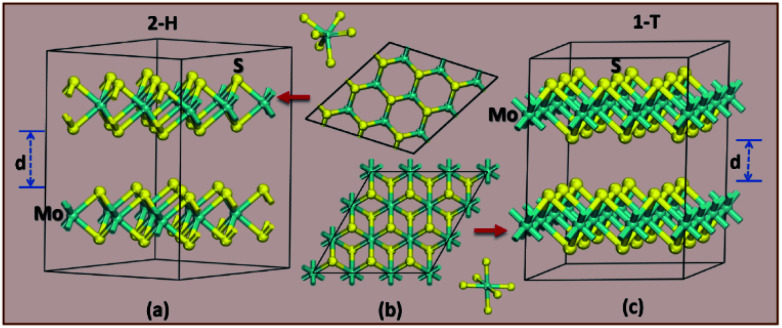
The difference in crystal structure for 2H (a) and 1T (c) MoS_2_ polymorphs. (b) Shows a top-down look on the hexagonal polymorph of 2H (top) and 1T (bottom).

The structural stability of the several different phases of MoS_2_ has been studied to find the most stable phase and polymorph for further investigation and research. Our first step was to perform a total energy calculation as a function of volume for all the phases. Based on this calculation we divided the phases into two different groups (according to the energetics), group A and group B. The polymorphs in group A, shown in Fig. 1a, are (space group and space group number are given in the parenthesis): 2R_1_-MoS_2_ (*P*3*m*1; 156), 2H-MoS_2_ (*P*6_3_/*mmc*; 194), 3H_b_-MoS_2_ (*P*6_3_/*mmc*; 194), 4T-MoS_2_ (*P*3̄*m*1; 164), 3H_a_-MoS_2_ (*P*6̄*m*2; 187), 2T-MoS_2_ (*P*3̄*m*1; 164), and 1H-MoS_2_ (*P*6̄*m*2; 187). In group B, as shown in Fig. 1b, we have placed the following polymorph models: 1T_1_-MoS_2_ (*P*3̄; 164), 1T_2_-MoS_2_ (*P*3̄*m*1; 164), 3T-MoS_2_ (*R*3̄*m*; 166) and 2R_2_-MoS_2_ (*P*3̄*m*1; 164). It should be noted that the 2H and 3R variants the Mo–S coordination is trigonal prismatic and the layers stacking sequence are significantly different (see Fig. 2).^37^ On the other hand, the 1T variants consist of MoS_2_ layers with almost perfectly ordered MoS_6_ octahedra.^37^

**Fig. 3:**
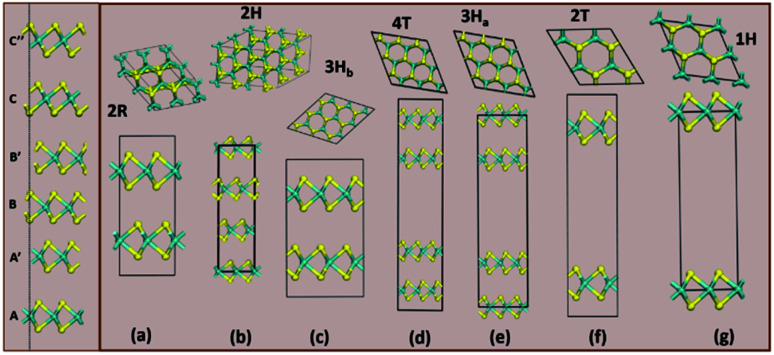
The column on the left shows the various stacking sequences (A, A′, B, B′, C, C′′) for MoS_2_. On the right side, we see how the group A polymorphs are stacked.

As shown in Fig. 1, the total energy curves clearly show that group A is energetically favoured over group B with an energy difference of 0.8 eV. In general, we see that our first principle calculations coincide well with experimental results.^38–41^ Interestingly, we observe in Fig. 1a that the various polymorphs in group A seem to have the same minimum energy, although with a varying range of volume. Which indicates that MoS_2_ can easily be found in any of these variants. The calculated positional and lattice constants of different polymorphs are presented in Table 1. From the space group numbers and names, we see that all group B polymorphs and three of the group A polymorphs are trigonal, while the last four group A polymorphs are hexagonal. From Fig. 1 it is clear that the hexagonal polymorphs have a wider spread in volume than the trigonal polymorphs. However, the involved energy difference in group A is small, and it is hard to conclude whether trigonal or hexagonal polymorphs are more energetically favourable. Regarding the group B polymorphs, three of them are trigonal crystal systems and of them 3T-MoS_2_ has the lowest energy. Another point of interest is how the volume affects the energy of the unit cell. For group A there is little difference between the energies and all the polymorphs could be synthesised (based on Fig. 1). However, for group B it appears that the two larger polymorphs (with regards to volume) are more energy favourable compared to the smaller ones.

Nonetheless, Fig. 1 only gives an indication of which polymorphs MoS_2_ prefers to be in, which is why we calculated the elasticity constants and phonon densities.

**Table 1:** Polymorph and lattice parameters for the investigated polymorphs

Polymorph	Cell constants (Å)	Coordinates
2R_1_-MoS_2_ (*P*3*m*1; 156)	*a* = 3.1887, *b* = 3.1887, *c* = 21.3444	Mo_1_ (1*a*): 0, 0, 0
		Mo_2_ (1*a*): 2/3, 1/3, 1/3
		Mo_3_ (1*a*): 1/3, 2/3, 2/3
		S1 (1*a*): 0, 0, 0.5928
		S2 (1*a*): 2/3, 1/3, 0.9271
		S3 (1*a*): 1/3, 2/3, 0.2604
		S4 (1*a*): 0, 0, 0.7400
		S5 (1*a*): 2/3, 1/3, 0.0733
		S6 (1*a*): 1/3, 2/3, 0.4067
2T-MoS_2_ (*P*3̄*m*1; 164)	*a* = 3.1891, *b* = 3.1891, *c* = 24.8987	Mo (2*d*): 1/3, 2/3, 0.8505
		S1 (2*d*): 1/3, 2/3, 0.2122
		S2 (2*d*): 1/3, 2/3, 0.0868
4T-MoS_2_ (*P*3̄*m*1; 164)	*a* = 3.1889, *b* = 3.1889, *c* = 39.7944	Mo_1_ (2*d*): 1/3, 2/3, 0.0936
		Mo_2_ (2*d*): 1/3, 2/3, 0.7193
		S1 (2*d*): 1/3, 2/3, 0.3199
		S2 (2*d*): 1/3, 2/3, 0.9457
		S3 (2*d*): 1/3, 2/3, 0.2414
		S4 (2*d*): 1/3, 2/3, 0.8672
1H-MoS_2_ (*P*6̄*m*2; 187)	*a* = 3.1881, *b* = 3.1881, *c* = 17.4639	Mo (1*a*): 0, 0, 0
		S (2*h*): 1/3, 2/3, 0.0894
3H_a_-MoS_2_ (*P*6̄*m*2; 187)	*a* = 3.1890, *b* = 3.1890, *c* = 32.3461	Mo_1_ (2*h*): 1/3, 2/3, 0.7698
		Mo_2_ (1*e*): 2/3, 1/3, 0
		S1 (2*h*): 1/3, 2/3, 0.0483
		S2 (2*i*): 2/3, 1/3, 0.2785
		S3 (2*i*): 2/3, 1/3, 0.8181
3H_b_-MoS_2_ (*P*6_3_/*mmc*; 194)	*a* = 3.1890, *b* = 3.1890, *c* = 14.8916	Mo (2*d*): 2/3, 1/3, 1/4
		S (4*f*): 2/3, 1/3, 0.8549
2H-MoS_2_ (*P*6_3_/*mmc*; 194)	*a* = 3.1779, *b* = 3.1779, *c* = 14.1156	Mo (2*b*): 0, 0, 1/4
		S (4*f*): 2/3, 1/3, 0.3608
2R_2_-MoS_2_ (*P*3̄*m*1; 164)	*a* = 3.1798, *b* = 3.1798, *c* = 6.5738	Mo (1*b*): 0, 0, 1/2
		S (2*d*): 2/3, 1/3, 0.2575
3T-MoS_2_ (*R*3̄*m*; 166)	*a* = 3.2060, *b* = 3.2060, *c* = 19.7232	Mo (3*a*): 1/3, 2/3, 2/3
		S (6*c*): 0, 0, 0.2534
1T_1_-MoS_2_ (*P*3̄; 164)	*a* = 3.1900, *b* = 3.1900, *c* = 5.9450	Mo (1*a*): 0, 0, 0
		S (2*d*): 1/3, 2/3, 0.2488
1T_2_-MoS_2_ (*P*3̄*m*1; 164)	*a* = 3.1900, *b* = 3.1900, *c* = 5.9450	Mo (1*a*): 0, 0, 0
		S (2*d*): 1/3, 2/3, 0.2488

### Band structure

**Fig. 4:**
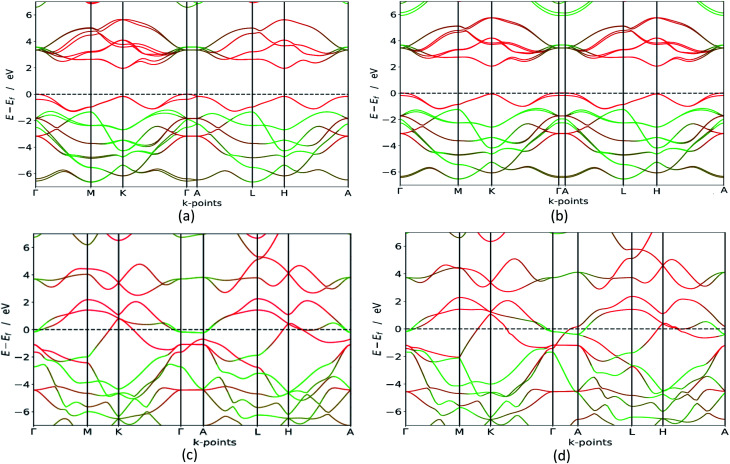
HSE06 band structure (colour code: green line – S, red line – Mo) for 3H_b_ in (a), 1H in (b), 2R_2_ in (c) and 1T_1_ in (d). We see that the group A polymorphs are semiconductors with a bandgap between 1.8 and 2.1 eV, while the group B polymorphs are metallic. The other polymorphs are seen in the ESI.†

In order to verify which of these polymorphs are viable for *e.g.* photocatalytic processes, photovoltaic cells or in transistors we carry out in depth electronic calculations. Materials with semiconducting properties could be used to absorb visible light, while metals could be used as conductors. Our HSE06 bandgap calculations, presented in Fig. 4, clearly states that the group B polymorphs are metallic, which is in line with previous findings.^42^ However, the polymorphs in group A are semiconductors with indirect bandgaps as the valence band is at the *Γ* point, while the conduction band minimum is although accurate in its band polymorph description, underestimates the bandgap value. GGA calculations are less accurate than HSE06 (ref. 43 and ^44^) but they are cheaper in computing time, making them excellent for first-time investigations and gives an idea about the bandgap configuration. This is confirmed when the GGA results are compared to the HSE06 calculations, which are also presented in Table 2. Our HSE06 results coincide well with the experimentally found bandgaps for MoS_2_ which are within the range of 1.2–1.9 eV.^7^ The valence bands and conduction bands for the polymorphs in both groups are derived from Mo-d and S-p states.^21^ This shows that the group A MoS_2_ is well suited for photovoltaic solar cell and photocatalytic water splitting applications.

**Table 2:** Calculated GGA and HSE06 total bandgaps (*E*_g_; in eV), type of bandgap, the effective mass of electrons and effective mass of holes. The effective masses are calculated along the *K*–*Γ K*-path

Name	GGA band gap (eV)	HSE06 band gap (eV)	The effective mass of electrons 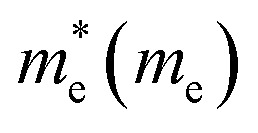 in *K*–*Γ* directions	Effective mass of holes 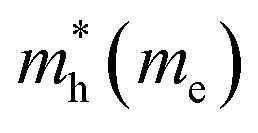 in *K*–*Γ* direction	Effective mass of hole 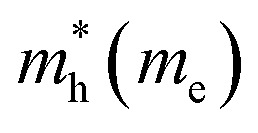 in *Γ*–*A* and *Γ*–*M* directions	Type of bandgap
2R_1_-MoS_2_	1.41	1.87	0.50	0.57	0.92 (*Γ*–*A*)	Indirect
2H-MoS_2_	1.42	1.94	0.51	0.55	0.86 (*Γ*–*A*)	Indirect
3H_b_-MoS_2_	1.45	1.95	0.22	0.03	1.01 (*Γ*–*A*)	Indirect
4T-MoS_2_	1.48	1.96	0.48	0.56	1.82 (*Γ*–*M*)	Indirect
3H_a_-MoS_2_	1.50	1.98	0.47	0.56	1.89 (*Γ*–M)	Indirect
2T-MoS_2_	1.54	2.04	0.47	0.56	2.32 (*Γ*–*M*)	Indirect
1H-MoS_2_	1.64	2.12	0.47	0.56	2.96 (*Γ*–*M*)	Indirect
MoS_2_ (ref. 48)	1.58 (LDA)	2.48 (*G*_0_*W*_0_)	0.55	0.53	NA	NA

The electron effective mass is an indication of the mass of the structure/particle when it responds to forces. It can be used to calculate electron mobility and diffusion constants. We used Fonari and Sutton's effective mass calculator for our calculations.^45^ The higher curvature of the conduction band minimum compared to the valence band minimum indicates a higher hole effective mass than the electron effective mass. This indicates that MoS_2_ has higher electron mobility, compared to the hole mobility, due to the lower electron effective mass.

We calculated the effective masses for the semiconductor (group A) polymorphs to confirm the findings in the band structures. In general, the effective masses of electrons and holes are relevant for the mobility, electrical resistivity, quantum confinement,^46,47^ and free-carrier optical response in semiconductor materials. For the first time, effective masses are presented for seven different polymorphs of MoS_2_ and are shown in Table 2. We have compared them to a 2H-MoS_2_ polymorph from Rasmussen *et al.* to get an indication of how our polymorphs measure up against previously studied polymorphs, and we see that our values are lower for electrons.^48^ This is due to the different approximations (*G*_0_*W*_0_) used in the calculations.

For photocatalytic processes, the transfer of carriers to the reactive sites is easier with smaller effective masses.^49^ Compared to 2H-TiO_2_ (1.4*m*_e_ and 5*m*_h_)^48^ and 1T-TiO_2_ (8.2*m*_e_ and 1.1*m*_h_)^48^ the electron mobility in MoS_2_ is better than that of TiO_2_. This combined with a much lower bandgap (3.2 eV for TiO_2_ (ref. 50)) clearly show that MoS_2_ is a better photocatalyst than TiO_2_.

Further research on carrier transport characteristics is needed as the presence of valleys and defects in the polymorph, charge carrier scattering, reduced mean free path and elastic scattering time all influence the carrier mobility in the crystal.

### Phonon calculations

**Fig. 5:**
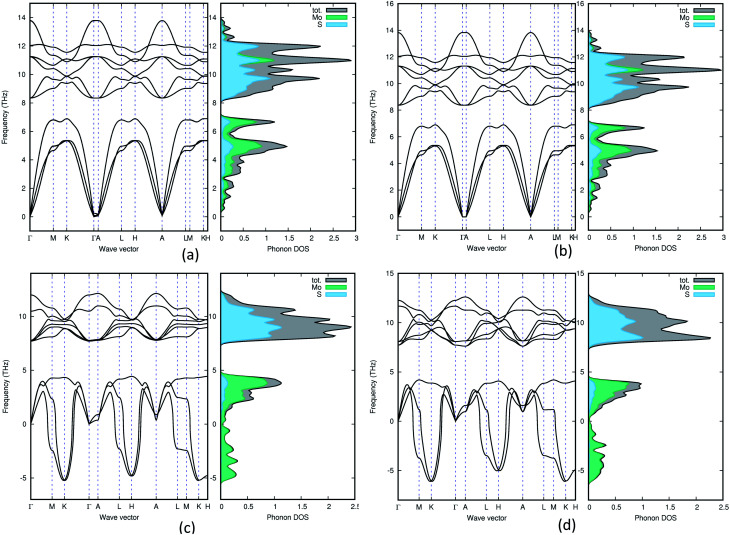
Phonon density of states for 3H_b_ (a), 1H (b), 2R_2_ (c) and 1T_1_ (d). Both group B polymorphs (2R_2_ and 1T_1_) contains negative frequencies, which means that they are dynamically unstable.

In order to understand the dynamical stability of the studied polymorphs we carried out phonon calculations. In addition to the total phonon density of states (PDOS), we calculated the phonon dispersion curves, at the equilibrium volume, along the high symmetry direction of the Brillouin zone for all the polymorphs and these variations are presented in Fig. 5 with their corresponding PDOS. None of the group A polymorphs displays any soft/negative modes, which means that they should be dynamically stable. Whereas the group B polymorphs show the presence of negative modes, making them dynamically unstable. This shows that going from 2H polymorphs to 1T polymorphs creates a less stable polymorph, which is supported by experimental findings.^51^

The total phonon density of states is calculated at the equilibrium volumes for the different polymorphs of MoS_2_. From Fig. 5 we observe that the two group B polymorphs (all four can be found in SI 4a–d†) contains unstable (imaginary) phonon modes while for the two group A (SI 3a–g† for the remaining polymorphs) polymorphs we only have stable (real) modes. These findings indicate that the group A polymorphs are dynamically stable, while the group B polymorphs are dynamically unstable. All group A polymorphs have a similar PDOS, this combined with the low energy difference between phases indicates that one can easily modify one polymorph into another using temperature or pressure. This explains why depending on different synthesis routes it is possible to stabilise different MoS_2_ polymorphs.^7^ Not surprisingly we find that 1T_2_-MoS_2_ and 1T_1_-MoS_2_ have very similar wave vectors, PDOS and partial PDOS, as they are both trigonal and share the same lattice parameters (see Table 1) although they are in different space groups. Comparing 3T-MoS_2_ to 2R_2_-MoS_2_ there is a slight difference in where the maximum peaks are, this could be explained by the difference in the volume of the unit cell. For group A, they all seem quite similar, except for 2H-MoS_2_ which have a slightly different distribution in the higher frequency area compared to the others. Indicating that it has fewer occupied states in the 11 THz regions compared to the others.

The partial PDOS are included in Fig. 5 as well and it is clear that the smaller atom S dominates the higher frequencies (above 8 THz), while the heavier Mo atom dominates the lower frequencies. However, some S modes appear in the low-frequency region and for the 2H polymorphs, a few Mo modes appear above 10 THz.

### Mechanical stability

We have computed the single-crystal elastic constants to help us understand the mechanical stability of the investigated MoS_2_ phases. The elastic constants of a material describe how the material responds to an applied force, as either applied strain or the required stress to maintain a certain deformation. Both stress and strain have three tensile and three shear components. Due to this, the elastic constants of a crystal can be described using a 6 × 6 symmetric matrix, having 27 components where 21 of those are independent. Naturally, we can reduce the number of components by utilising any existing symmetry in the polymorph. The 6 × 6 matrix is known as *C*_*ij*_, the stiffness matrix, and it can be used to calculate properties as the bulk modulus, Poisson coefficient and Lame constants. Previous studies show that the accuracy of the DFT elastic constant is within 10% of the experimental values.^63^ Hence, we can safely use our results to predict the elastic constant for our MoS_2_ polymorphs.

For trigonal polymorphs the mechanical stability criteria of the elastic constants are:^64^*B*_T1_ = *C*_11_ − *C*_12_ > 0*B*_T2_ = (*C*_11_ + *C*_12_)*C*_33_ > 2*C*_13_^2^*B*_T3_ = (*C*_11_ − *C*_12_)*C*_44_ > 2*C*_14_^2^*B*_T4_ = *C*_44_ > 0

For the hexagonal polymorphs the stability criteria are:^64^*B*_H1_ = *C*_11_ >|*C*_12_|*B*_H2_ = (*C*_11_ + *C*_12_)*C*_33_ > 2*C*_13_^2^*B*_H3_ = *C*_44_ > 0*B*_H4_ = *C*_66_ > 0

As seen in Table 3, only 1T_2_-MoS_2_ is found to be mechanically unstable since it does not fulfil the Born criteria. Even though group B polymorphs fulfil the Born criteria this does not imply that these could be synthesised as they were found to be dynamically unstable based on the phonon analysis. In general, if a compound is found to be dynamically stable, it indicates that it has either a stable phase or a possible metastable phase. All A group materials are both dynamically and mechanically stable, so these polymorphs can be synthesised experimentally. Since the B group materials are dynamically unstable, but mechanically stable (except 1T_2_-MoS_2_) we could conclude that these polymorphs have metastable phases. This explains why monovalent elements/nanoparticles/nanoobjects have been added to stabilise group B polymorphs.^29,65–67^

**Table 3:** The calculated single-crystal elastic constants *C*_*ij*_ (in GPa), bulk modulus *B* (in GPa), shear modulus *G* (in GPa), Poisson's ratio *ν*, Young's modulus *E* (in GPa). Subscript V indicates the Voigt bound, R indicates the Reuss bound and H indicates the Hill bound

Polymorph	2R_1_-MoS_2_	2T-MoS_2_	4T-MoS_2_	1H-MoS_2_	3H_a_-MoS_2_	3H_b_-MoS_2_	2H-MoS_2_	2R_2_-MoS_2_	3T-MoS_2_	1T_1_-MoS_2_	1T_2_-MoS_2_
Crystal system	Trigonal	Trigonal	Trigonal	Hexagonal	Hexagonal	Hexagonal	Hexagonal	Trigonal	Trigonal	Trigonal	Trigonal
*C*_11_		105	132	75	123	176	190	140	177	187	195
*C*_12_		27	34	19	31	45	48	8	−4	37	44
*C*_13_		0.1	0.40	0.1	0.5	0.6	2	10	14	30	58
*C*_14_		0	0	0	0	0	0	0	0	0	0
*C*_33_		0.3	1	0.3	1	2	7	14	29	10	12
*C*_44_		39	49	28	0.4	66	71	66	90	75	75
*C*_66_		0.2	0.42	0.1	46	0.3	0.21	4	6	31	−82
Born		Yes	Yes	Yes	Yes	Yes	Yes	Yes	Yes	Yes	No
*B*_V_		29	37	21	35	50	55	39	48	64	80
*B*_R_		0.3	1	0.3	1	2	7	14	26	4	−125
*B*_H_		15	19	11	18	26	31	26	37	34	−23
*G*_V_		20	72	14	24	34	37	32	44	104	−2
*G*_R_		0.4	1	0.2	1	0.8	1	4	12	5	−41
*G*_H_		10	36	7	12	17	19	18	28	55	−21
*ν*_V_		0.22	−0.09	0.22	0.22	0.22	0.23	0.18	0.15	−0.03	0.51
*ν*_R_		0.08	0.19	0.25	0.23	0.35	0.46	0.36	0.30	0.01	0.35
*ν*_H_		0.22	−0.08	0.22	0.22	0.23	0.25	0.22	0.20	−0.02	0.14
*E*_V_		49	131	35	58	83	90	76	102	203	−5
*E*_R_		0.9	2	0.5	2	2	2	11	31	10	−110
*E*_H_		25	66	18	30	43	47	45	68	106	−48

To investigate how the polymorphs would react to applied mechanical forces, we calculated the Voigt (V), Reuss (R) and Hill (H) modulus through the elastic stiffness moduli, *C*_*ij*_. These were then used to calculate the bulk modulus B, shear modulus *G*, Young's modulus *E* and Poisson's ratio *ν*. The calculated values are found in Table 3.

The Hill average young modulus for 1T_2_-MoS_2_ (−48 GPa) is negative, which indicates that the atoms are stretched instead of being compressed. For 2T-MoS_2_ (25 GPa), 4T-MoS_2_ (66 GPa), 1H-MoS_2_ (18 GPa), 3H_a_-MoS_2_ (30 GPa), 3H_b_-MoS_2_ (43 GPa), 2H-MoS_2_ (47 GPa), 2R_2_-MoS_2_ (45 GPa), 3T-MoS_2_ (68 GPa) and 1T_1_-MoS_2_ (106 GPa) the atoms are compressed due to the positive value. We see that there is spread in the stiffness of the polymorphs varying from 1H-MoS_2_ with 18 GPa (like peptide nanotubes^68,69^) up to 1T_1_-MoS_2_ at 106 GPa (like bronze, brass and some titanium alloys^70^).

Looking at the Poisson's ratio, we see that 4T-MoS _2_ and 1T_1_-MoS_2_ have negative values, −0.08 and −0.02, which makes them auxetic materials. This means that when the materials are subjected to a positive strain along a longitudinal axis, the transverse strain would increase the cross-sectional area. MoS_2_ is known for being among crystalline materials that have polymorphs with negative Poisson's ratio,^71^ and 1T polymorphs are the more common auxetic polymorphs.^72^ Auxetic materials are expected to have mechanical properties such as high energy absorption and fracture resistance.

The other materials vary from a Poisson's ratio of 0.14 (1T_2_-MoS_2_) up to 0.25 (2H-MoS_2_), which is a range from foam-like compressibility to cast iron. The average of our polymorphs seems to be 0.2, which is around cast iron. In addition to Youngs' modulus and Poisson's ratio, we can also calculate shear modulus over bulk modulus (*G*/*B*), a value that will determine if the material is ductile or brittle. The critical value for high (low) *G*/*B* that separates ductile and brittle materials is 0.5.^73^ Our calculated *G*/*B* values are below 0.5, implying that all the polymorphs have brittle characteristics except 3H_a_-MoS_2_ which has a *G*/*B* value of 0.97. 3H_a_-MoS_2_ is thus expected to be a ductile material.

### Raman and IR spectra

#### IR spectrum

**Fig. 6:**
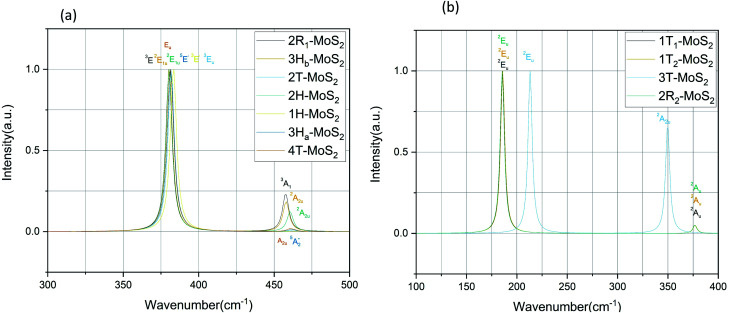
IR spectra for the group A polymorphs (a) and the group B polymorphs (b).

The IR spectra of all the studied MoS_2_ polymorphs are presented in Fig. 6, and the corresponding modes are presented in Table 4. From the calculated values, we clearly observe that the high frequency modes are caused by S–Mo–S rotation, whereas low frequency modes are caused by Mo–S vibrations. According to crystal symmetry, A_2u_ and E_1u_ IR modes refer to a bulk material, while 
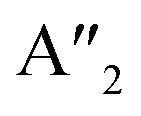
 plus E′ correspond to single layer, and A_2u_ and E_u_ are active IR modes for double layer MoS_2_.^52^ Based on the calculated IR spectra for the group B polymorphs shown in Fig. 6b, we see that 3T-MoS_2_ is a double-layer polymorph (due to comparatively larger intermediate distance between the layers), while 1T_1_-MoS_2_ and 1T_2_-MoS_2_ contain the ^2^E_u_ from double-layer polymorphs in addition to much softer ^2^A_u_ mode. Our results clearly show that the group B polymorphs are only metastable, and this may the reason for lack of other theoretical IR studies in the literature on these polymorphs. This makes it difficult to verify this result due to lack of literature data. Further theoretical and experimental studies are needed on this aspect.

**Table 4:** The calculated Raman and IR frequency (in cm^−1^) for the modes at the *Γ* point of the Brillouin zone for MoS_2_ polymorphs

Polymorph	Raman active modes	IR active modes
2R_1_-MoS_2_	^3^E: 286, 381. ^3^A_1_: 405	^3^E: 380. ^3^A_1_: 457
2H-MoS_2_	^2^E_2g_: 30, 382. ^1^E_1g_: 283. ^1^A_1g_: 404	^2^E_1u_: 380. ^2^A_2u_: 460
3H_b_-MoS_2_	^2^E_2g_: 36, 380. ^1^E_1g_: 284. ^1^A_1g_: 403	^2^E_1u_: 380. ^2^A_2u_: 458
4T-MoS_2_	E_g_: 14, 33, 282, 283, 380. A_1g_: 22, 53, 401, 403, 461, 463	E_u_: 26, 281, 283, 380. A_2u_: 43, 399, 402, 463
3H_a_-MoS_2_	^4^E′′: 19. ^5^E′: 283, 381. 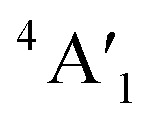 : 398, 461	^5^E′: 381. 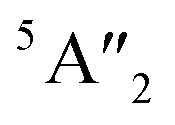 : 461
2T-MoS_2_	^3^A_1g_: 40, 400. ^3^E_g_: 284, 382	^3^E_u_: 382
1H-MoS_2_	^1^E′′: 284. ^2^E′: 383	^3^E′: 384
1T_1_-MoS_2_	^1^E_g_: 274. ^1^A_1g_: 386	^2^E_u_: 186. ^2^A_u_: 377
1T_2_-MoS_2_	^1^E_g_: 275. ^1^A_1g_: 386	^2^E_u_: 186. ^2^A_u_: 377
3T-MoS_2_	^1^E_g_: 258. ^1^A_1g_: 398	^2^E_u_: 213. ^2^A_2u_: 350
2R_2_-MoS_2_	^1^E_g_: 274. ^1^A_1g_: 386	^2^E_u_: 185. ^2^A_u_: 376
Bulk 2H-MoS_2_	E^1^_2g_: 384^a^, 382^b^, 384^c^. A_1g_: 408^a^, 408^b^, 408^c^	E_1u_: 382^e^, 384^f^, 384^g^. A_2u_: 468^f^, 470^g^
Mono 2H-MoS_2_	E′: 384^d^, 385^c^. 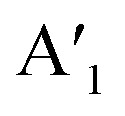 : 403^d^, 404^c^	

a From ref. 58.

b From ref. 59.

c From ref. 60.

d From ref. 49.

e From ref. 61.

f From ref. 62.

g From ref. 54.

Regarding the group A polymorphs, we clearly notice the presence of ^2^E_1u_ and ^2^A_2u_ active modes for 3H_b_-MoS_2_ and 2H-MoS_2_ indicating that they are MoS_2_ bulk polymorphs. 4T-MoS_2_ has E_u_ and A_2u_ as active modes, which is also an indication of a bulk polymorph. Due to the presences of the E′ and 
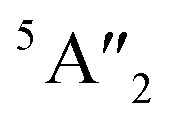
 modes (due to comparatively larger intermediate distance between the layers) we find 3H_a_-MoS_2_ to be a single layer. The E_u_ modes seen for 2T-MoS_2_ confirms that this a double layer polymorph, while the E′ mode for 1H-MoS_2_ makes it a single layer polymorph. 2R_1_-MoS_2_ on the other hand shows E modes and A_1_, neither of these modes have previously been reported as active IR modes for MoS_2_. This could be an artefact from the calculation method, although the historical known accuracy speaks against this. However, it could also be a result of the interlayer distance and van der Waals forces making it harder to differentiate between the MoS_2_ layers of the polymorph. Another possible explanation is that the polymorphs are tilted slightly, and therefore exist in a state between 2H and 1T. This would change the crystal symmetry enough to introduce previously unseen modes.

#### Raman spectra

**Fig. 7:**
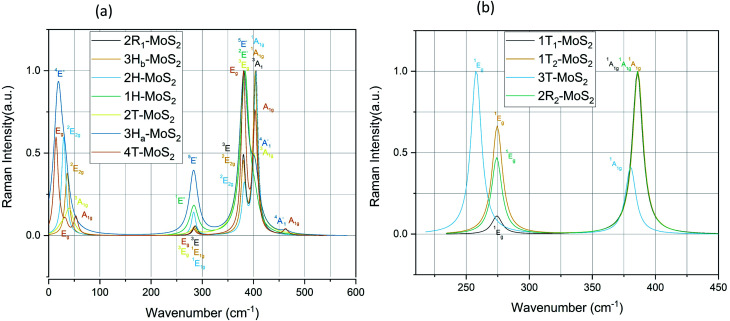
Raman spectra for the group A polymorphs (a) and the group B polymorphs (b).

All of our polymorphs exhibit the signature Raman active modes E^1^_g_ and A_1g_,^53^ as shown in Fig. 7 and Table 4. In group B polymorphs, out-of-plane ^1^A_1g_ mode is dominant, which indicates single degenerate wave functions, except for 3T-MoS_2_ which is dominated by the in-plane ^1^E_g_ mode. Compared to the modes of 3T-MoS_2_ we see that the modes of the other polymorphs are redshifted. The observed redshift could be attributed to the larger interlayer distances (a factor of almost 4, see Table 1). This could lead to an increase in the dielectric screening of the long-range Coulomb forces and thus reduce the overall restoring force on the atoms. From Fig. 7, we observe that the group A polymorphs have a widespread in dominating modes compared to group B. The E^1^_g_, E^2^_2g_ and A_1g_ modes around 280 cm^−1^, 380 cm^−1^ and 410 cm^−1^ are in agreement with experimental studies.^54,55^ The modes seen at the lower end of Fig. 7 (<100 cm^−1^) arise from the vibration of an S–Mo–S layer against adjacent layers, while E^1^_2g_ stems from opposite vibration of two S atoms with respect to the Mo atom. In general, the A_1g_ mode is associated with the out-of-plane vibrations of only S atoms in opposite directions. The additional 
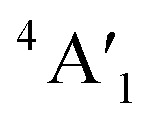
 mode (∼460 cm^−1^) for 3H_a_-MoS_2_ are due to strong electron–phonon couplings and could come from a second-order process involving the longitudinal acoustic phonons at *M* point (LA(*M*)).^56^ We also note that the E^1^_g_ and A_1g_ are redshifted compared to the Raman modes of group B polymorphs. Raman spectra can be used to verify the crystallinity of a material. The Raman spectra for crystalline materials contain sharper peaks or long-range correlations, while amorphous materials only have short-range ordering.^57^ Raman spectra indicates clearly that the MoS_2_ polymorphs considered in this study are shown to have crystalline characteristics.

For the sake of checking the validity of our approach, we have tabulated experimental as well as other theoretical findings on 2H-MoS_2_ polymorph. Based on our knowledge, there are still no studies reported on 1T polymorphs due to the synthesis and stability challenges of these polymorphs. We see that in general, we have the same major peaks around 380 cm^−1^ and 405 cm^−1^ for group A polymorphs as reported in the literature. The same is observed with the IR modes, which are in good agreement with reported literature data.

## Conclusion

For the very first time 14 different MoS_2_ polymorphs are proposed and studied using DFT total-energy calculations, band structure analysis, phonon density of states and elastic constants calculations. The in-depth study shows.

• Three of the polymorphs were omitted from the study because their energy-volume data were far away from the data for other polymorphs, which indicates that these polymorphs are unstable.

• Polymorphs in group B (1T_1_-MoS_2_, 1T_2_-MoS_2_, 3T-MoS_2_ and 2R_2_-MoS_2_) are all metallic and lacked dynamical stability. 1T_2_-MoS_2_ is neither dynamical stable nor mechanical stable.

• Group A (2R_1_-MoS_2_, 3H_b_-MoS_2_, 2H-MoS_2_, 1H-MoS_2_, 2T-MoS_2_, 3H_a_-MoS_2_ and 4T-MoS_2_) polymorphs are semiconductors with an indirect bandgap, the range for the seven polymorphs is 1.87 eV to 2.12 eV. They are all dynamically and mechanically stable.

• 2R_1_-MoS_2_ has the lowest bandgap of 1.87 eV.

• 4T-MoS_2_ stands out due to being auxetic, which means it has a high level of fracture resistance.

• 3H_b_-MoS_2_ has the lowest effective electron mass (0.22*m*_e_*vs.* for example 1.4*m*_e_ for 2H-TiO_2_, which is widely used in PV and photocatalytic applications).

Our theoretical analysis show that the candidates in group A can be readily synthesised. Here further experimental verification is needed. The bandgap range of 1.87 eV to 2.12 eV makes the group A polymorphs viable for photovoltaic and photocatalytic applications. Out of the seven polymorphs in group A, 3H_b_-MoS_2_, with its high electron mobility and with the bandgap of 1.95 eV, is the most promising candidate for photovoltaic and photocatalytic applications. MoS_2_ has recently shown promise as electron and/or hole-transport layer in perovskite solar cells, and the high carrier mobility of 3H_b_-MoS_2_ makes it a promising candidate for this use.

The group B polymorphs were only found to be metastable phases (except 1T_2_-MoS_2_) and cannot be synthesised. Due to the transitions of metastable phases in 1T polymorphs, more research on these polymorphs is needed such that the synthesis of a pure 1T-MoS_2_ single-layer polymorph is viable.

## Conflicts of interest

There are no conflicts of interest to declare.

## Supplementary Material

RA-011-D0RA10443D-s001

RA-011-D0RA10443D-s002

RA-011-D0RA10443D-s003

RA-011-D0RA10443D-s004

RA-011-D0RA10443D-s005

RA-011-D0RA10443D-s006

RA-011-D0RA10443D-s007

RA-011-D0RA10443D-s008

RA-011-D0RA10443D-s009

RA-011-D0RA10443D-s010

RA-011-D0RA10443D-s011

RA-011-D0RA10443D-s012
