# Correction: In-depth first-principle study on novel MoS_2_ polymorphs

**DOI:** 10.1039/d1ra90092g

**Published:** 2021-03-25

**Authors:** Håkon Eidsvåg, Murugesan Rasukkannu, Dhayalan Velauthapillai, Ponniah Vajeeston

**Affiliations:** Department of Computing, Mathematics and Physics, Western Norway University of Applied Sciences Inndalsveien 28, Box 5063 Bergen Norway heid@hvl.no; Center for Materials Science and Nanotechnology, Department of Chemistry, University of Oslo Box 1033 Blindern N-0315 Oslo Norway

## Abstract

Correction for ‘In-depth first-principle study on novel MoS_2_ polymorphs’ by Håkon Eidsvåg *et al.*, *RSC Adv.*, 2021, **11**, 3759–3769, DOI: 10.1039/D0RA10443D.

The authors regret that there was an error in the sentences from line 24 in the right column on page 3759 to line 3 in the left column on page 3760 of the original article. The text originally read, “Early research suggests that bulk MoS_2_ could metallize under pressure as they found that the bandgap shrinks due to a negative pressure coefficient of resistivity, d*E*_G_/d*P* < 0.^16^ Unfortunately, the structural transition is unknown, and it requires further research.” These sentences should read, “For example, if the pressure is above 25 to 35 GPa it will cause a structural transition from 2H_c_-MoS_2_ to 2H_a_-MoS_2_ as the MoS_2_ layers will slide in a process similar to superlubric sliding.^16^ This will also result in band overlap metallization in both structures.^16^”

The Royal Society of Chemistry apologises for these errors and any consequent inconvenience to authors and readers.

## Supplementary Material

