# Supplementary material for: In-depth first-principle study on novel MoS2 polymorphs
Source: RSC Adv. 2021 Jan 19;11(6):3759–69. doi: 10.1039/d0ra10443d (PMC8694157; doi:10.1039/d0ra10443d)
Supplement: RA-011-D0RA10443D-s004 [file RA-011-D0RA10443D-s004.pdf]

Electronic Supplementary Material (ESI) for RSC Advances.
This journal is © The Royal Society of Chemistry 2021

Novel in-depth first-principle stability study of 2H and 1T MoS2 polymorphs supplementary information

Håkon Eidsvåg*[a], Murugesan Rasukkannu [a], Dhayalan Velauthapillai [a] and Ponniah Vajeeston[b]

Electronic structure:

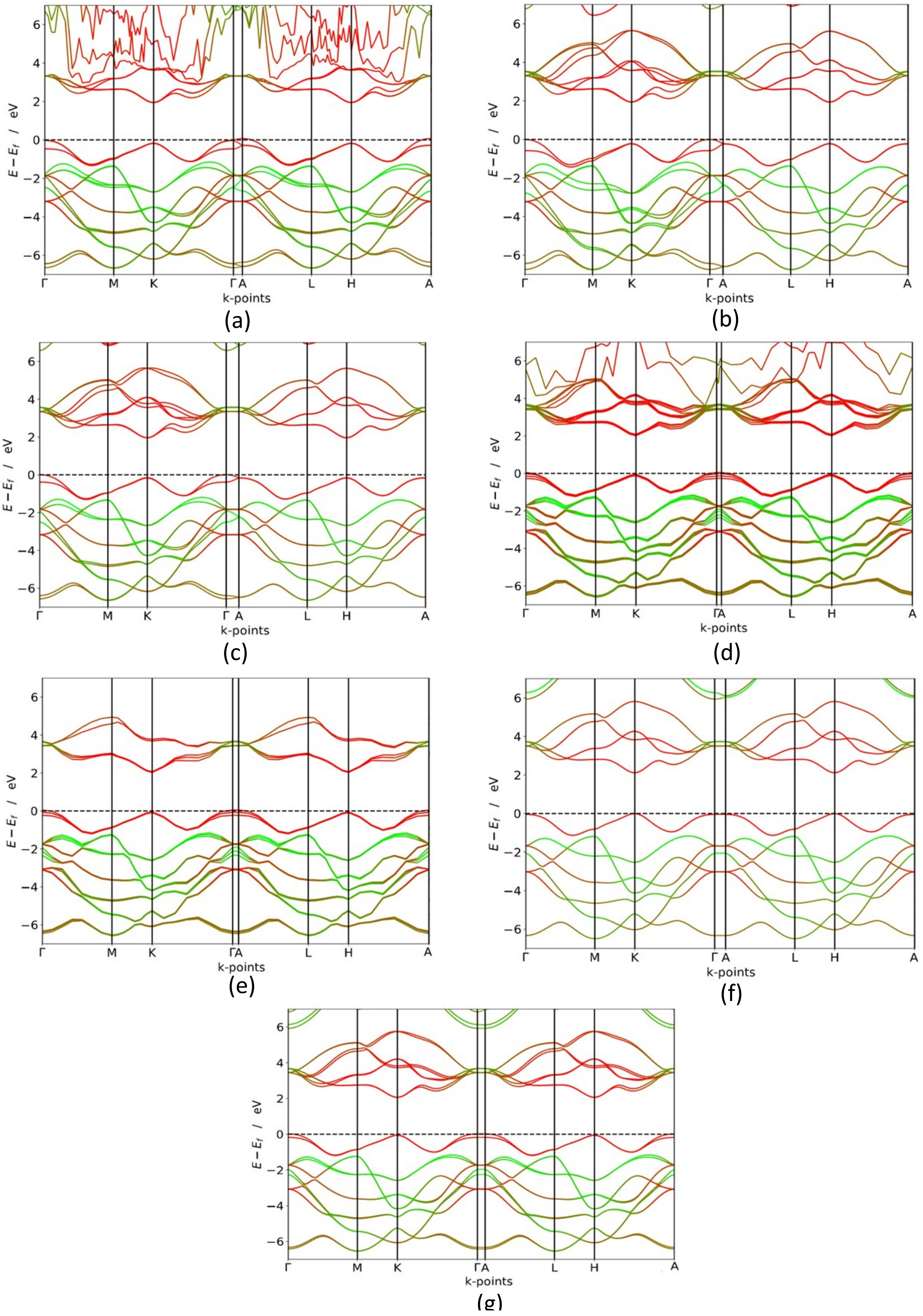

SI 1: Band structure for group A polymorphs (Colour code: green line – S, red line – Mo). (a) $2R_1$. (b) 2H. (c) $3H_b$. (d) 4T. (e) $3H_a$. (f) 2T. (g) 1H

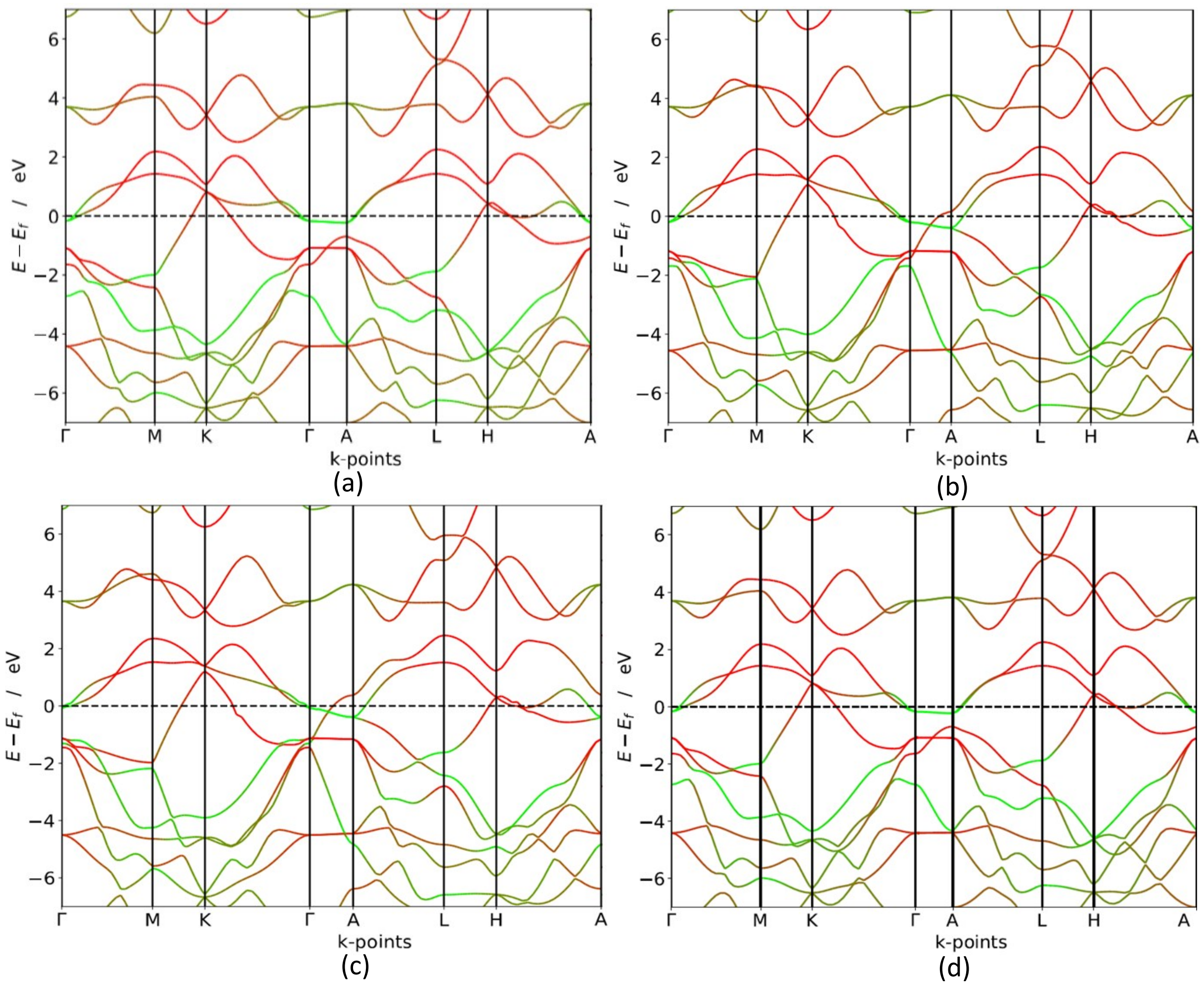

SI 2: Band structure for the group B polymorphs (Colour code: green line – S, red line – Mo). (a) $2R_2$. (b) $1T_1$. (c) $1T_2$. (d) 3T.

Phonon DOS and band structures

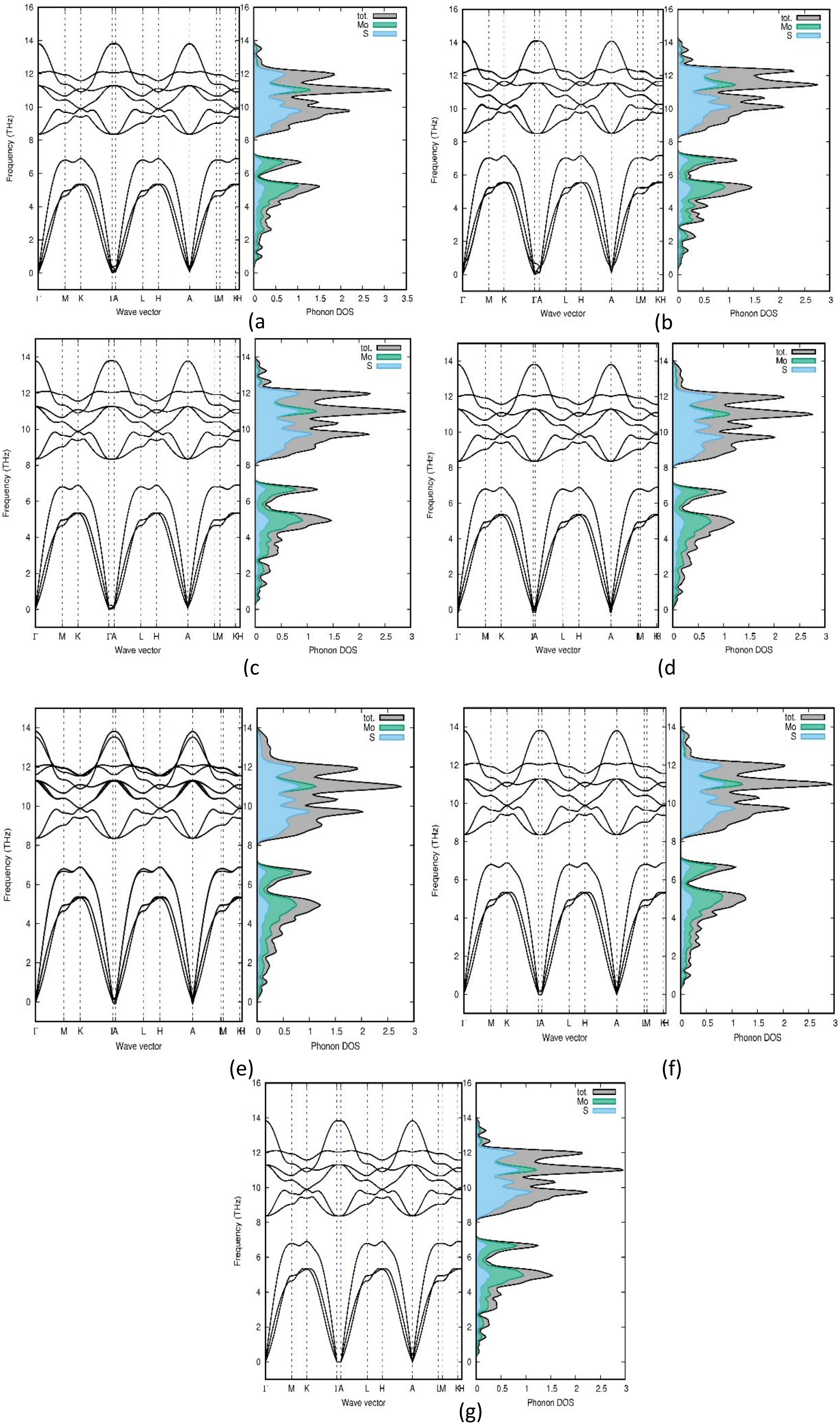

SI 3: Phonon density of states calculations for the group A polymorphs. (a) $2R_1$. (b) 2H. (c) $3H_b$. (d) 4T. (e) $3H_a$. (f) 2T. (g) 1H.

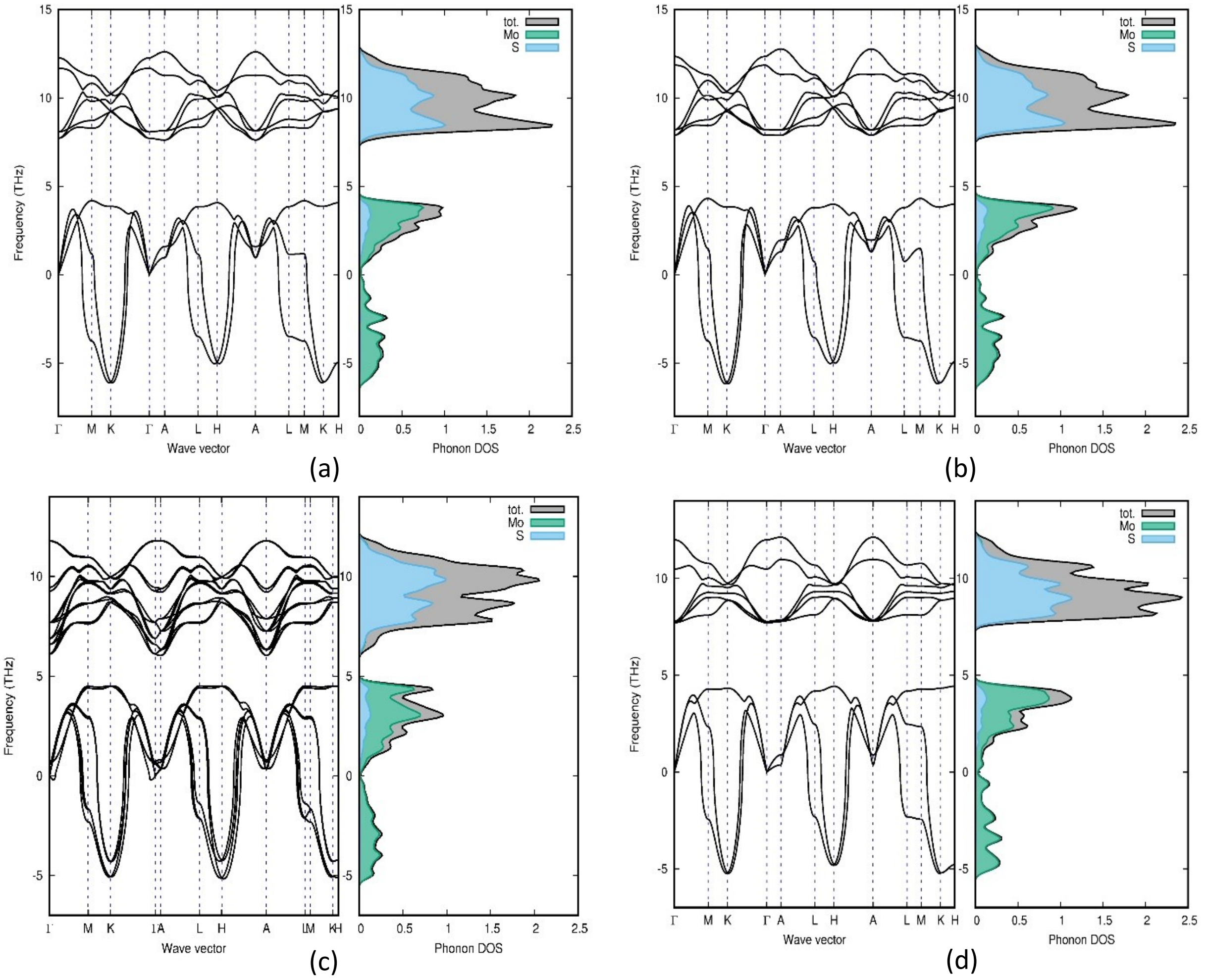

SI 4: Phonon density of states calculations for the group B polymorphs. (a) $1T_1$. (b) $1T_2$. (c) 3T. (d) $2R_2$.

Optimised structures:

Poscar:

***R3m* (1, 1434, 2R$_1$),**

```
Mo3 S6
1.0
        7.3491001129         0.0000000000         0.0000000000
        6.6573448829         3.1127209286         0.0000000000
        6.6573448829         1.4794943865         2.7386361459
   Mo     S
    1     2
Direct
     0.000200000         0.000200000         0.000200000
     0.593800008         0.593800008         0.593800008
     0.740000010         0.740000010         0.740000010
```

***P-3m1* (4, 1027525, 4T),**

```
 Mo4 S8
 3.18890000
     0.86602540    -0.50000000     0.00000000
```

```
     0.00000000     1.00000000     0.00000000
     0.00000000     0.00000000    12.47903666
Mo  Mo  S  S  S  S
   2 2 2 2 2 2
Direct
        0.33333333    0.66666667    0.09356000
        0.66666667    0.33333333    0.90644000
        0.33333333    0.66666667    0.71932000
        0.66666667    0.33333333    0.28068000
        0.33333333    0.66666667    0.31991000
        0.66666667    0.33333333    0.68009000
        0.33333333    0.66666667    0.94568000
        0.66666667    0.33333333    0.05432000
        0.33333333    0.66666667    0.24145000
        0.66666667    0.33333333    0.75855000
        0.33333333    0.66666667    0.86720000
        0.66666667    0.33333333    0.13280000
```

P63/mmc (2, 2815, **2H**);

```
Mo2 S4
1.0
        3.1889998913         0.0000000000         0.0000000000
       -1.5944999456         2.7617549185         0.0000000000
        0.0000000000         0.0000000000        14.8915996552
   Mo    S
    2    4
Cartesian
     1.594499993        0.920585000         3.722899914
     0.000000000        1.841169836        11.168699741
     1.594499993        0.920585000        12.730828582
     0.000000000        1.841169836         2.160771073
     0.000000000        1.841169836         5.285028755
     1.594499993        0.920585000         9.606570901
```

P-6m2 (3, 1025874, **3H$_a$**),

```
Mo3 S6
 3.18900000
     0.86602540    -0.50000000     0.00000000
     0.00000000     1.00000000     0.00000000
     0.00000000     0.00000000    10.14302289
  Mo  S Mo  S  S
   2 2 1 2 2
Direct
        0.33333333    0.66666667    0.76980000
        0.33333333    0.66666667    0.23020000
        0.33333333    0.66666667    0.04827000
        0.33333333    0.66666667    0.95173000
        0.66666667    0.33333333    0.00000000
        0.66666667    0.33333333    0.27846000
        0.66666667    0.33333333    0.72154000
        0.66666667    0.33333333    0.81807000
        0.66666667    0.33333333    0.18193000
```

P-3m1 (3, 1023939, **2T**),

```
Mo2 S4
```

```
 3.18910000
     0.86602540    -0.50000000     0.00000000
     0.00000000     1.00000000     0.00000000
     0.00000000     0.00000000     7.80743784
  Mo S S
    2 2 2
Direct
        0.33333333    0.66666667    0.85049000
        0.66666667    0.33333333    0.14951000
        0.33333333    0.66666667    0.21222000
        0.66666667    0.33333333    0.78778000
        0.33333333    0.66666667    0.08682000
        0.66666667    0.33333333    0.91318000
```

P63/mmc (6, 1018809, **3H$_b$**),

```
Mo2 S4
1.0
        3.1779000759         0.0000000000         0.0000000000
       -1.5889500380         2.7521421964         0.0000000000
        0.0000000000         0.0000000000        14.1155996323
   Mo    S
    2    4
Direct
     0.000000000         0.000000000         0.250000000
     0.000000000         0.000000000         0.750000000
     0.666666687         0.333333343         0.360799998
     0.333333313         0.666666627         0.639199972
     0.333333313         0.666666627         0.860800028
     0.666666687         0.333333343         0.139200002
```

P-6m2 (1, 1023924, **1H**),

```
Mo S2
 3.18810000
     0.86602540    -0.50000000     0.00000000
     0.00000000     1.00000000     0.00000000
     0.00000000     0.00000000     5.47783947
  Mo S
    1 2
Direct
        0.00000000    0.00000000    0.00000000
        0.33333333    0.66666667    0.08937000
        0.33333333    0.66666667    0.91063000
```

R-3m (1, 558544, **3T**),

```
Mo S2
 3.20600000
     0.57735027     0.00000000     2.05065502
    -0.28867513     0.50000000     2.05065502
    -0.28867513    -0.50000000     2.05065502
    Mo S
  1 2
Direct
        0.00000000    0.00000001    0.00000000
        0.25339000    0.25339000    0.25339000
```

```
        0.74661000    0.74661000    0.74661000
```

P-3m1 (1, 1238797, **$2R_2$**),

```
Mo S2
 3.17980000
     0.86602540    -0.50000000     0.00000000
     0.00000000     1.00000000     0.00000000
     0.00000000     0.00000000     2.06736273
    Mo  S
  1  2
Direct
        0.00000000    0.00000000    0.50000000
        0.33333333    0.66666667    0.25751000
        0.66666667    0.33333333    0.74249000
```

P-3 (1, **$1T_2$**)

```
Mo S2
 3.19000000
     0.86602540    -0.50000000     0.00000000
     0.00000000     1.00000000     0.00000000
     0.00000000     0.00000000     1.86363636
    Mo  S
  1  2
Direct
        0.00000000    0.00000000    0.00000000
        0.33333333    0.66666667    0.24880000
        0.66666667    0.33333333    0.75120000
```

P-3m1 (1, **$1T_1$**)

```
Mo S2
 3.19000000
     0.86602540    -0.50000000     0.00000000
     0.00000000     1.00000000     0.00000000
     0.00000000     0.00000000     1.86363636
    Mo  S
  1  2
Direct
        0.00000000    0.00000000    0.00000000
        0.33333333    0.66666667    0.24880000
        0.66666667    0.33333333    0.75120000
```
